# Sucupira Oil-Loaded Nanostructured Lipid Carriers (NLC): Lipid Screening, Factorial Design, Release Profile, and Cytotoxicity

**DOI:** 10.3390/molecules25030685

**Published:** 2020-02-06

**Authors:** Raquel Vieira, Patricia Severino, Luciana A. Nalone, Selma B. Souto, Amélia M. Silva, Massimo Lucarini, Alessandra Durazzo, Antonello Santini, Eliana B. Souto

**Affiliations:** 1 Department of Pharmaceutical Technology, Faculty of Pharmacy (FFUC), University of Coimbra, Pólo das Ciências da Saúde, Azinhaga de Santa Comba, 3000-548 Coimbra, Portugal; raquelvieira.fmuc@gmail.com; 2 Laboratory of Nanotechnology and Nanomedicine (LNMED), Institute of Technology and Research (ITP), Av. Murilo Dantas, 300, 49010-390 Aracaju, Brazil; pattypharma@gmail.com (P.S.); luciana.nalone@hotmail.com (L.A.N.); 3 Industrial Biotechnology Program, University of Tiradentes (UNIT), Av. Murilo Dantas 300, 49032-490 Aracaju, Brazil; 4 Institute for Technology and Research (ITP), Tiradentes Institute, 150 Mt Vernon St, Dorchester, MA 02125, USA; 5 Department of Endocrinology, Hospital de São João, Alameda Prof. Hernâni Monteiro, 4200-319 Porto, Portugal; sbsouto.md@gmail.com; 6 Department of Biology and Environment, University of Trás-os-Montes e Alto Douro, UTAD, Quinta de Prados, P-5001-801 Vila Real, Portugal; amsilva@utad.pt; 7 Centre for Research and Technology of Agro-Environmental and Biological Sciences, CITAB, UTAD, Quinta de Prados, P-5001-801 Vila Real, Portugal; 8 CREA—Research Centre for Food and Nutrition, Via Ardeatina 546, 00178 Rome, Italy; massimo.lucarini@crea.gov.it (M.L.); alessandra.durazzo@crea.gov.it (A.D.); 9 Department of Pharmacy, University of Napoli Federico II, Via D. Montesano 49, 80131 Napoli, Italy; 10 CEB—Centre of Biological Engineering, University of Minho, Campus de Gualtar, 4710-057 Braga, Portugal

**Keywords:** essential oil, sucupira oil, diabetes mellitus, nanostructured lipid carriers (NLC), hot HPH, cytotoxicity

## Abstract

Essential oils are odorant liquid oily products consisting of a complex mixture of volatile compounds obtained from a plant raw material. They have been increasingly proven to act as potential natural agents in the treatment of several human conditions, including diabetes mellitus (DM). DM is a metabolic disorder characterized by chronic hyperglycemia closely related to carbohydrate, protein and fat metabolism disturbances. In order to explore novel approaches for the management of DM our group proposes the encapsulation of sucupira essential oil, obtained from the fruits of the Brazilian plants of the genus *Pterodon*, in nanostructured lipid carriers (NLCs), a second generation of lipid nanoparticles which act as new controlled drug delivery system (DDS). Encapsulation was performed by hot high-pressure homogenization (HPH) technique and the samples were then analyzed by dynamic light scattering (DLS) for mean average size and polydispersity index (PI) and by electrophoretic light scattering (ELS) for zeta potential (ZP), immediately after production and after 24 h of storage at 4 °C. An optimal sucupira-loaded NLC was found to consist of 0.5% (*m*/*V*) sucupira oil, 4.5% (*m*/*V*) of Kollivax^®^ GMS II and 1.425% (*m*/*V*) of TPGS (formulation no. 6) characterized by a mean particle size ranging from 148.1 ± 0.9815 nm (0 h) to 159.3 ± 9.539 nm (at 24 h), a PI from 0.274 ± 0.029 (0 h) to 0.305 ± 0.028 (24 h) and a ZP from −0.00236 ± 0.147 mV (at 0 h) to 0.125 ± 0.162 (at 24 h). The encapsulation efficiency and loading capacity were 99.98% and 9.6%, respectively. The optimized formulation followed a modified release profile fitting the first order kinetics, over a period of 8 h. In vitro cytotoxicity studies were performed against Caco-2 cell lines, for which the cell viability above 90% confirmed the non-cytotoxic profile of both blank and sucupira oil-loaded NLC.

## 1. Introduction

Essential oils (EOs) are odorant liquid oily products, consisting of a complex mixture of lipophilic and volatile compounds obtained from a plant raw material by one of three distinct procedures: Dry distillation, driving by steam of water or suitable mechanical method without heating [1,2,3,4,5,6]. Among their most abundant constituents, terpenes, sesquiterpenes and flavonoids are receiving increasing attention as potential natural agents for the treatment of several human diseases [7,8,9,10,11,12,13,14,15] (e.g., rheumatoid arthritis and other inflammatory diseases, cardiovascular diseases, diabetes and metabolic syndrome [16,17,18]). Among chronic diseases, diabetes mellitus (DM) is recognized the epidemy of the 21st century. It is characterized by chronic hyperglycemia together with disturbances in carbohydrate, protein, and fat metabolism, resulting from an imbalance between insulin availability and insulin need [15,19,20,21]. There are two main types of DM. DM type 1 or insulin-dependent diabetes mellitus (T1DM or IDDM) represents about 10% of the cases and results from the destruction of insulin-secreting pancreatic β-cells by an autoimmune-mediated process. DM type 2 or non-insulin-dependent diabetes mellitus (T2DM or NIDDM) occurs in the remaining 90% of the cases and is characterized as a chronic and progressive metabolic disorder of both carbohydrate and lipid metabolism. T2DM is characterized by the presence of hyperglycemia, mainly due to peripheral insulin resistance, and/or by enhanced hepatic glucose production associated with impaired glucose homeostasis [22]. In DM, depending on the disease stage, the patient can show either higher, normal or low insulin levels resulting from an impaired β-cell function and insulin secretion, and also from impaired hepatic glucose production [23]. Subcutaneous injections of insulin cause discomfort, pain, trauma, being also associated with the risk of local infections, hypoglycemia, skin necrosis, and nerve damage [24,25]. This administration route compromises patient’s compliance, especially in elderly population [26,27].

Sucupira essential oil is obtained from the fruits of the Brazilian plants of the genus *Pterodon*, commonly known as “faveira”, “sucupira”, “sucupira-branca”, “fava-de-sucupira”, or “sucupira-lisa” and has been recognized as promising agent for the management of DM and its complications [17,28]. Traditional medicine has early recognized its diverse biological properties, (e.g., anti-nociceptive, anti-inflammatory, antioxidant, antimicrobial, anticancer, muscle relaxant, hypoglycemic, and lipolytic activities) [18]. These are mainly due to the presence of isoflavones, sesquiterpenes and diterpenes in the composition of the oil, being linear and/or tetracyclic diterpenes with a vinhaticane or vouacapane skeleton the most abundant ones [16]. However, some difficulties are still encountered in the delivery of EOs to their targets in vivo, due to their inherent high lipophilicity and high volatility [3,16,29]. Furthermore, as they are very sensitive to oxygen, light, moisture and heat, EOs are highly sensitive to degradation, namely oxidation, producing many free oxygen radical species and other oxidation products, such as lipid hydroperoxides, aldehydes, hydrocarbons, ketones, and epoxides, thus losing their main biological activity [3,16,29].

To overcome the above-mentioned issues and to benefit from their biological activities, nanoparticles have been proposed to load EOs for therapeutic use [7,9,30]. Nanoparticles have the advantages of site-specific targeted delivery of bioactive molecules, improving their bioavailability with minimal toxicological events [29,31]. Among different types of nanoparticles, solid lipid nanoparticles (SLN) and nanostructured lipid carriers (NLC) have a special interest due to their lipid composition and solid matrix [32]. The selected lipids (e.g., triacylglycerols, fatty acids, steroids, waxes, or oils) are physiological, biocompatible and biodegradable in vivo, and act as absorption enhancers thereby increasing the bioavailability of the payload [33,34,35]. By definition, SLN are composed only of solid lipids, either highly purified triglycerides, complex glyceride mixtures or waxes, stabilized in aqueous dispersion by a surfactant. These particles are suitable for the loading of poorly-water soluble drugs and lipophilic compounds, and shown high physical stability, protection of the loaded bioactive against degradation, controlled release and high tolerability both in vivo and in vitro [36,37,38,39]. Over shelf-life, these particles may however undergo polymorphic changes which can compromise their loading capacity for certain drugs, resulting in drug expulsion from the particles. Attempting to overcome this, NLC were designed composed of a mixture of solid and liquid lipids covered by a surfactant layer in aqueous dispersion. The blend of solid and liquid lipids delays or prevents polymorphic changes of the solid lipid during crystallization and enhanced stability [40,41,42,43]. NLC are specially tailored for the delivery of lipophilic compounds [44].

This work aimed to formulate sucupira essential oil in NLC, optimizing the nanoparticle formulation by a 2^2^ factorial design experiment after selecting the appropriate solid lipid by a lipid screening assay. The mean particle size, polydispersity index (PI) and zeta potential (ZP) were set as dependent variables, and the effect of lipid and surfactant concentrations (independent variables) evaluated. The optimized sucupira-NLC formulation was then characterized for its loading capacity, encapsulation efficiency, release kinetics, and cytotoxic profile in Caco-2 cell lines as in vitro model for oral drug delivery.

## 2. Material and Methods

### 2.1. Materials

#### 2.1.1. Lipids

Sucupira essential oil was obtained from the fruits of genus *Pterodon* plant and used as liquid lipid. Imwitor^®^ 900 K (glycerol monostearate, type II) with a monoglyceride content of 40–55% (m/m) and Dynasan^®^ 116 (tripalmitin) were purchased from Cremer Oleo GmbH & Co. KG company (Hamburg, Germany) and used as solid lipid. Kollivax^®^ GMS II (glycerol monostearate) was purchased from BASF (Ludwigshafen am Rhein, Germany) and used as solid lipid. Compritol^®^ 888 ATO (glyceryl dibehenate) was purchased from Gattefossé (Lyon, France). Cetostearyl alcohol was purchased from Sigma-Aldrich (Madrid, Spain) and used as solid lipid.

#### 2.1.2. Surfactants

TPGS (also known as d-α-Tocopherol polyethylene glycol succinate, vitamin E polyethylene glycol succinate or vitamin E-TPGS) and Poloxamer 188 (also known as Kolliphor^®^ P188) were purchased from BASF (Ludwigshafen am Rhein, Germany). Tween^®^ 80 (also known as polysorbatum 80, polysorbate 80 or polyoxyethylene sorbitan monooleate) was purchased from Sigma-Aldrich (Madrid, Spain). 

#### 2.1.3. Other Materials

Soy lecithin (Phospholipon 80H, phosphatidylcholine hydrogenated) was purchased from Lipoid GmbH (Cologne, Germany). Ultra-purified water was obtained from a MilliQ Plus system (Millipore, Germany).

### 2.2. Methods

#### 2.2.1. Lipid Screening

Lipid screening was performed as described by Doktorovova et al. [45], by mixing 5%, 10%, and 15% (*m*/*V*) of sucupira oil with each of the above-mentioned solid lipids at the concentrations 95%, 90%, and 85% (*m*/*V*), respectively. The obtained fifteen samples of 1 *g* were heated in an incubator at 95 °C for 30 min (above the melting point of the selected solid lipids). The mixtures where visually checked every 15 min up to an hour, and then after 24 h to check the miscibility of both lipids when melted and after full recrystallization of the solid lipid (i.e., 24 h). The same procedure was repeated for samples which demonstrated no phase separation to choose the best sucupira oil–solid lipid ratio.

#### 2.2.2. Surfactant Screening

Surfactant screening was performed by ultrasonication. Firstly, six samples of 50 mL with different contents in TPGS, Tween 80, Poloxamer 188, Lecithin and ultrapurified water, as illustrated in Table 1, were mixed on a stirring plate at 500 rpm, without heating, for 10 min. Each sample was then submitted to a Vibra-Cell™ ultrasonic processor (VCX750-220V, Sigma-Aldrich, Sintra, Portugal) at maximum amplitude, with no pulse, for 2 min. Secondly, six other samples of 50 mL containing different amounts of TPGS, Tween 80, Poloxamer 188, Lecithin, ultrapurified water, sucupira oil and Kollivax^®^ GMS II were submitted to the same procedure. 

#### 2.2.3. Preparation of Sucupira Oil-Loaded NLC

Sucupira oil-loaded NLC were produced by hot high-pressure homogenization (HPH) technique, as described by Souto et al. [46]. The lipid phase consisting of sucupira oil (0.50 or 0.75% (*m*/*V*)) and either the solid lipid Imwitor^®^ 900 K (4.50; 4.25% (*m*/*V*)) or Kollivax GMS II^®^ (4.5 or 4.25% (*m*/*V*)) was dispersed in an aqueous phase consisting of TPGS (0.475, 0.950, or 1.425% (*m*/*V*)) and ultrapurified water (94.525, 94.050, or 93.575% (*m*/*V*)) (Table 2), at 68 °C (approximately 5 °C above the melting point of the solid lipid). The final volume of all nine formulations was 50 mL. Each of the obtained heated coarse pre-emulsions were processed by high-shear mixing Ultra-Turrax T25 (Ystral GmbH D-7801, Dottingen, Germany) at 16,000 rpm for 15 min, followed by hot HPH (Emulsiflex^®^-C3, Avestin, Inc., Ottawa, Canada) operating continuously for 20 min with a set pressure of 600 bar.

#### 2.2.4. Physicochemical Characterization: Size, Polydispersity Index, and Zeta Potential Analysis

Samples were analyzed by dynamic light scattering (DLS) for particle size and polydispersity index (PI), immediately after production and 24 h after. Both particle size and PI were determined by using a Zetasizer Nano ZS (Malvern, Worcestershire, UK), which has a 0.3 nm to 10 μm particle size range and was equipped with a laser beam (λ = 633 nm; 4 mW) and a scattered light detector positioned at an angle of 173° (non-invasive backscatter) in order to unmask scattered light signals of low intensity originated by the smaller particles [42]. All samples underwent a 100 times dilution in ultrapurified water, and particle size and PI measurements were made in disposable cells at 25 °C and analyzed in triplicate measurements (n = 3) (33 runs per measurement). In turn, ZP was determined by electrophoretic light scattering (ELS) at 25 °C by using a Zetasizer Nano ZS (Malvern, Worcestershire, UK). The samples were also diluted 100 times in ultra-purified water and analyzed in triplicate measurements (n = 3) (30 runs for measurement). The Henry’s equation with the Smoluchowski approximation was used for ZP calculation. Data were expressed as arithmetical means ± standard deviations (SD).

#### 2.2.5. Encapsulation Efficiency (EE) and Loading Capacity (LC)

To calculate the encapsulation efficiency (EE) and loading capacity (LC) of sucupira oil in NLC, particles were firstly ultra-centrifuged for 1 h at 100,000× *g* in a Beckman Optima™ Ultracentrifuge (Optima™ XL, Indianapolis, IN) and the quantification of all-trans (−)-14,15-epoxygeranyl-geraniol, the main component of *Pterodon* sp. [47], was determined in the supernatant. The sample was analyzed in a UV spectrophotometer Shimadzu UV-1601 (Shimadzu Italy, Cornaredo, Italy) at 290 nm [48], and EE and LC determined applying the following equations [34]:

(1) $$EE\left(\%\right)=\frac{W_{a}-w_{s}}{w_{a}}\times 100$$

(2) $$LC\left(\%\right)=\frac{W_{a}-w_{s}}{w_{a}-w_{s}+w_{L}}\times 100$$

where *W_a_* is the weight of sucupira oil added for the production, *W_L_* is the weight of lipid added for the production and *W_s_* is the weight of sucupira oil in the supernatant.

#### 2.2.6. Experimental Factorial Design

Aiming to maximize the experimental efficiency with a minimum number of experiments to optimize the sucupira oil-loaded NLC produced by HPH, a full factorial design approach gathering all the possible combinations between the factors and their levels was applied. A 2^2^ factorial design consisting of two factors, each one set at 2-levels [49], was used to evaluate surfactant (TPGS) and solid lipid (Kollivax^®^ GMS II) concentrations influence on the produced NLC. As dependent variables, mean particle size, PI and ZP were considered. For each factor, the lower and higher values of the lower and upper levels were represented by (−1) and (+1), respectively. The central point, represented by (0), was replicated three times to estimate the experimental error (Table 3). Data was analyzed by the STATISTICA 7.0 software (Stafsoft, Inc., Tulsa, OK, USA). NLC dispersions were randomly produced and the analysis of variance, ANOVA statistical test, was performed for each response parameter to identify effects’ significance and their interactions. A *p* value < 0.05 was considered statistically significant.

#### 2.2.7. In Vitro Release Profile

The in vitro release profile of sucupira oil from NLC was assessed using Franz glass diffusion cells, designed with a donor and an acceptor chamber. Before the assay, a cellulose membrane with an average pore size of 0.22 µm (MERCK KgaA) was soaked for a few hours in freshly prepared phosphate-buffered saline (PBS, pH7.4), and then placed in between the two chambers. A volume of 1 mL of the optimized NLC dispersion was placed onto the hydrated cellulose membrane. The acceptor chamber, containing 5 mL of PBS buffer, was kept at 37 °C over the course of the assay under magnetic stirring. At pre-determined time-intervals, a volume of 200 µL was sampled with a syringe, being the same volume replaced with PBS buffer to ensure sink conditions. The amount of released sucupira oil was analyzed in a UV spectrophotometer Shimadzu UV-1601 (Shimadzu Italy, Cornaredo, Italy) at 290 nm. For the mathematical fitting, Four kinetic models, i.e., zero order, and first order kinetics, Higuchi and Korsmeyer-Peppas, have been used [50] for the mathematical fitting of the release profile, selecting the most appropriate based on the obtained R^2^ values. 

#### 2.2.8. In Vitro Cytotoxicity Studies

Caco-2 cell lines were purchased from Cell Lines Services (CLS, Eppelheim, Germany) and were used for the evaluation of the cytotoxicity of NLC. Cells were kept in Dulbecco’s Modified Eagle Medium (DMEM), containing 25 mM glucose, supplemented with 10% (*v*/*v*) fetal bovine serum (FBS; Gibco, Life Technologies, Thermo Fisher, Walton MA, United States), 2 mM L-glutamine, and antibiotics (100 U/mL penicillin and 100 μg/mL streptomycin), at 37 °C in controlled humidity atmosphere of 5% CO_2_ in air [33,51]. Cells were treated over time and in a concentration-dependent fashion, with blank NLC (non-loaded) and sucupira oil-loaded NLC formulations at different concentrations (5, 10, 15 and 20 µg/mL of solid lipid content), prepared in an aseptic laminar flow chamber. The supernatant of confluent cells was removed and cells were exposed at 37 °C for 10 min to trypsin until their complete detachment and disaggregation. Trypsin reaction was finalized with culture medium, cells were re-suspended, counted, seeded into 96-well microplates at a density of 5 × 10^4^ cells/mL (100 μL/well). Cells were cultured for 24 h. Culture media was then removed and replaced by FBS-free culture media supplemented with NLC formulations and incubated for additional 24 h. For estimation of the cell survival rate, 10% (*v*/*v*) Alamar Blue (Invitrogen Corporation, Carlsbad, CA, United States) was added to the medium, and absorbance was monitored (Multiskan EX, Labsystems, Midland, ON, Canada) at wavelengths 570 nm and 620 nm, after 4 h culture, as described by the manufacturer’s protocol, and after 24 and 48 h of exposure to test solution. Results were analyzed by determining the percentage of AB reduction, expressed as percentage of control (untreated cells), as described before [52].

#### 2.2.9. Statistics

Statistical analysis of data was performed using STATISTICA 7.0 (Stafsoft, Inc., Palo Alto, CA, United States) software, being a *p*-value < 0.05 accepted as significant [53]. Data were expressed as the mean value ± standard deviation (Mean ± SD), usually from three independent experiments (n = 3). Statistical analysis of in vitro cellular assays was performed by Student’s *t*-test (*p* < 0.05).

## 3. Results and Discussion

Since a mixture of a solid lipid (fat) with a liquid lipid (oil) form the lipid phase of the NLC emulsion, a lipid screening assay was firstly performed to select the best solid lipid for the production of NLC. For a successful NLC production, the selection of a proper lipid blend is very important, especially to ensure particle’s chemical stability to limit the risk of phase separation over storage time [7,30,54]. Thus, a suitable lipid blend requires (i) the solubility of the active compound (oil) in lipid matrix (solid lipid) to attempt lipid incorporation into lipid nanoparticles, ensuring proper drug loading capacity and encapsulation efficiency; (ii) a major spatial incompatibility of liquid and solid lipids molecules, wherein the oil molecules have no active participation in the solid crystalline matrix of the solid lipid and solid lipid crystals, in turn, are not dissolved in the oil; (iii) stability of lipid phase to chemical degradation; (iv) biodegradable lipids, also capable to produce nanometric particles; and (v) an acceptable toxicological profile, with no production of toxic residues along NLC production process [36,39]. For the lipid screening, visual checking of phase separation, within 24 h after melting the mixture of active ingredient and solid lipid, is a standard procedure [45,55,56,57]. The mixture of active ingredient and lipid has time enough to cool down so that the lipid recrystallizes. If no phase separation occurs, the active ingredient is solubilized in the recrystallized solid lipid and can therefore be selected for the production of the lipid nanoparticles [56]. When performing the visual analysis of the melted mixture (Table 4), if phase separation occurred, two yellow tonalities could be clearly identified, whereas only one single yellow tonality was observed if the lipid combination was miscible. It was expected that sucupira oil could be incorporated into the solid lipid core when either Imwitor^®^ 900 K (samples no. 1, 5, and 8), Dynasan^®^ 116 (samples no. 2 and 6), Kollivax^®^ GMS II (samples no. 3, 7, and 9), cetostearyl alcohol (sample no. 4) were used as solid lipids, at different concentrations. Compritol^®^ 888 ATO was not suitable to be used as solid lipid since a macroscopic phase separation was observed at a temperature below its melting point. We found out that lipid mixtures having sucupira oil and Imwitor^®^ 900 K or Kollivax^®^ GMS II may be used in sucupira oil-loaded NLC production since they create one single phase, even at different concentrations.

The next step was the choice of the surfactant. Usually, NLC formulations are preferentially composed of a single surfactant but, a combination of at least two surfactants is commonly reported, to improve the functional properties and to provide greater physical stability of the particles [58]. We have chosen three of the most used surfactants (Tween 80, Poloxamer 188 and Lecithin) and TPGS (a novel nonionic surfactant which exhibits amphipathic properties and allows the formation of stable micelles in aqueous media [58,59,60]). Tween 80, an oleate ester of sorbitol, is a water-soluble and non-ionic synthetic surfactant with a hydrophilic–lipophilic balance (HLB) value of ≈15 [61]. Poloxamer 188 is a copolymer that acts as a nonionic surfactant, with a HLB value of ≈29. Soy lecithin is a naturally occurring lecithin consisting of a complex mixture of essentially hydrophobic lipids, including phospholipids (namely phosphatidylcholine, phosphatidylethanolamine, and phosphatidylinositol), with an HLB value of ≈8, which makes lecithin only suitable for NLC stabilization when in combination with other surfactants. Additionally, functioning as a zwitterionic surfactant, lecithin may contribute either to a net negative, neutral or positive particle charge, depending on electrolyte concentration and pH [58]. Compared to ionic surfactants, non-ionic ones have demonstrated less toxicity and low irritation potential (REF).

Surfactant screening was performed by ultrasonication in a two-step approach and the results obtained from DLS and ELS analyses are depicted in Table 5. First, six samples (no. 1 to 6) with no lipid phase were produced in order to understand surfactant interactions that lead to smaller and more stable particles. Second, other six samples (no. 7 to 12) were produced with a solid lipid phase, attempting to evaluate the interaction between lipid and aqueous phases and also particle size and stability.

From the first step, formulation no. 1 has shown the smallest particle size immediately after production (197.8 ± 6.3 nm), remaining stable after 24 h (173.3 ± 2.9 nm), and with a PI nearly 0.3 (0.327 ± 0.008 arb units). It suggests that using together Tween 80, Poloxamer 188, TPGS and lecithin as aqueous phase, NLC may adopt a smaller size and increase stability. In turn, formulation no. 2 showed the smallest particle size after 24 h (21.4 ± 2.1 nm) with a PI of 0.242 ± 0.053 arb units, which may suggest that formulations with Tween 80, Poloxamer 188, TPGS but no lecithin take longer to stabilize but have some potential as aqueous phase. Finally, formulation no. 6 showed a particle size of nearly 200 nm and a PI of nearly 0.3 at both 0 h and 24 h after production, suggesting that an aqueous phase consisting of TPGS and Poloxamer^®^ 188 could be a good approach.

Regarding the second step, already with a lipid phase, all formulations were greater than 200 nm in size and formulations without the solid lipid (Kollivax^®^ GMS II) in its constitution got much more unstable. Immediately after production (0 h), formulation no. 12 showed the smallest particle size (243.6 ± 94.0 nm) and PI (0.351 ± 0.063 arb units) but after 24 h both parameters highly increased, thus suggesting that aqueous phase with only Tween^®^ 80 and lecithin may not be enough to encapsulate the oil. In turn, formulations no. 7 and no. 9 where the most stable ones with respect to particle size and PI values, although much higher than the expected for NLC formulations. It may suggest that even using only one surfactant in the aqueous phase (Tween^®^ 80 in both cases), a solid lipid may be integrated in the formulation since it stabilizes oil much more efficiently. These six samples should be previously heated at 68 °C for better solid and liquid phases interaction. Finally, for all twelve formulations ZP assumes a negative charge, which may be due to surfactant non-ionic character. Formulations no. 1, 3 and all those having a lipid phase achieved a ZP of at least −30 mV indicating electrostatic stabilization [58].

Since TPGS may be a good approach as surfactant, we decided to first produce sucupira-loaded NLC consisting of a lipid phase of sucupira oil (liquid lipid) and either Kollivax^®^ GMS II or Imwitor^®^ 900K as solid lipid, and an aqueous phase of a single surfactant, TPGS, as presented in Table 2. The results obtained from DLS and ELS measurements are represented in Table 6. 

In general, all samples achieved particle sizes and PI close to the expected for a NLC formulation, i.e., mean particle size ranging from 100 to 200 nm and PI < 0.3 arb units [36,47]. ZP assumes once more a negative charge or a value very close to zero, which may be due to surfactant non-ionic character.

Formulation no. 6 has shown the best results and greater stability, with a mean particle size ranging from 148.1 ± 1.0 nm (0 h) to 159.3 ± 9.6 nm (at 24 h) and a PI ranging from 0.274 ± 0.029 (0 h) to 0.305 ± 0.028 (24 h). Formulation no. 5 has also shown promising particle size range, from 164.8 ± 0.7 nm (0 h) to 178.1 ± 1.0 nm (at 24 h), although PI was greater than formulation no. 6. These findings may suggest that Kollivax^®^ GMS II is preferential over Imwitor^®^ 900K as solid lipid when NLC have a sucupira oil content of 0.5% (*m*/*V*). On the other hand, immediately after production formulations no. 8 and no. 9, having Imwitor^®^ 900K as solid lipid and encapsulating a greater sucupira oil amount (0.75% (*w*/*V*)), seemed to be very promising since it was obtained a mean particle size less than 150 nm and PI values less than 0.3 arb. units for both samples. However, after 24 h, both these values increased, with a mean particle size greater than 200 nm and a PI greater than 0.3 arb units. All sucupira oil-loaded NLC depicted a neutral surface charge (Table 6). The stability of NLC was ensured by the contribution of hydrophilic chains of TPGS (d-α-tocopheryl polyethylene glycol succinate). Indeed, stereochemical stabilization by the PEG chains surrounding the particles hinders their aggregation. This has been described for neutral nanoparticles formulated with hydrophilic surfactants (e.g., Tweens, Poloxamers) [7,62]. Besides, since the formulations are aimed to be administered orally, their use is not compromised by the PI as long as the particles remain physicochemically stable over shelf life until they are administered. 

Sucupira oil-loaded NLC were produced by hot HPH technique which, besides the advantages of smaller particle sizes and higher loading capacity for lipophilic bioactives, the appropriate combination of lipids and surfactants will have to be found. Experimental factorial design is well-established as a standard approach to reach the most suitable parameters allowing the production of stable sucupira oil-loaded NLC formulations [40,49,62,63,64,65,66]. Factorial design sets independent variables, either quantitative or qualitative factors, at given levels and evaluates them in a predefined number of experiments to understand their direct effect on the dependent variables and to determine if some factor interaction occurs. Solid lipid and surfactant concentrations were defined as factors and set at 2-levels each, with the lower and upper levels represented, respectively, by (−1) and (+1). Table 7 summarizes all seven experiments performed with reference to mean particle size, PI and ZP measurements performed 24 h after NLC production. Since, in the previous assay, after 24 h, formulation no. 5 showed to be the most stable, we selected it to be our pattern formulation in experimental factorial design. As such, a sucupira oil-loaded NLC with 0.5% (*m*/*V*) of sucupira oil, 4.5% (*m*/*V*) of Kollivax^®^ GMS II and 1.425% (*m*/*V*) of TPGS was defined as the central point (0) of the experiment and replicated three times (formulations no. 1 to 3, Table 7). Mean particle size ranged from 180.7 ± 11.8 nm (NLC no. 6) to 1762.0 ± 225.8 (NLC no. 4), PI in turn varied from 0.0613 ± 0.066 arb units (NLC n^o^.1) to 1.000 ± 0.000 arb units (NLC n^o^. 7) and, finally, ZP ranged from −0.0604 ± 0.0290 mV (NLC n^o^. 4) to +0.0757 ± 0.0425 mV (NLC n^o^. 6).

For each dependent variable—mean particle size, PI and ZP—an ANOVA was performed as summarized. A 95% confidence level (*p*-value = 0.05) was considered. None of the single factors, solid lipid and surfactant, nor their interaction had a statistically significant effect (*p*-value > 0.05) for either mean particle size, PI or ZP (Table 8, Table 9 and Table 10). However, slightly statistically significant effect (*p*-value of exactly 0.05) for ZP was observed when varying surfactant concentration.

**Figure 1:**
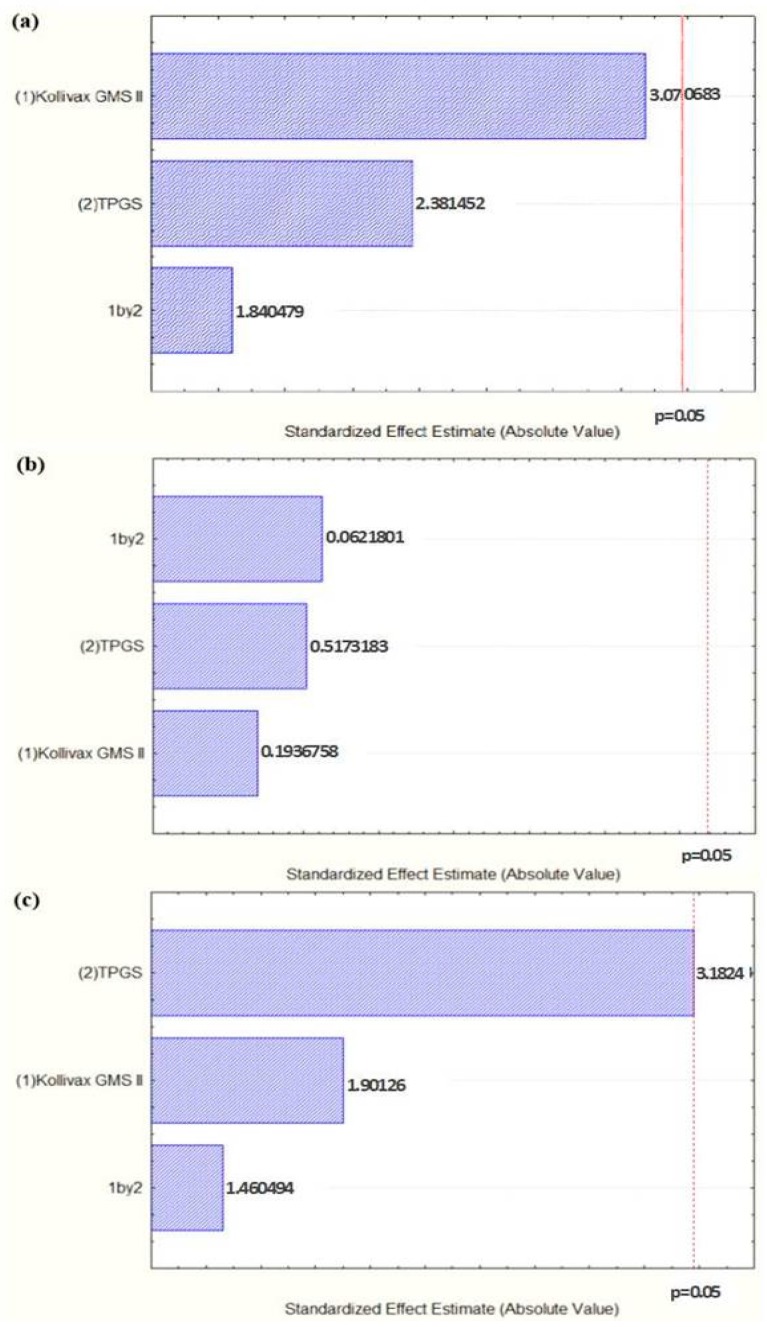
Pareto charts showing the effect of the concentration variation of the solid lipid (1), surfactant (2) and the interaction of both (1 by 2) on the sucupira oil-loaded NLC (**a**) mean particle size, (**b**) PI and (**c**) ZP.

Pareto charts, shown in Figure 1, set the *t*-value of effect and allow the study of response coefficients’ statistical significance. In Figure 1a, the variation of TPGS concentration from the lowest value to a high value had no significant prevailing positive effect on the particle size (*t*-value = 2.381452). The same may be observed in for PI (Figure 1b), with a *t*-value = 0.5173183. Controversially, for ZP (Figure 1c), varying TPGS concentration to a high value had a significant prevailing negative effect (*t*-value = −3.1824). Regarding either the variation of Kollivax^®^ GMS II concentration or the interaction between the variation of the surfactant and lipid ratio from the lowest value to a high one, they have shown no significant positive effect on particle size (Figure 1a) or PI (Figure 1b). Finally, for ZP (Figure 1c), the interaction between the variation of the surfactant and lipid ratio from the lowest value to a high one have shown no significant positive effect (*t*-value = 1.460494), and the variation of Kollivax^®^ GMS II concentration from the lowest value to a high value had no significant prevailing negative effect (*t*-value = −1.90126).

**Figure 2:**
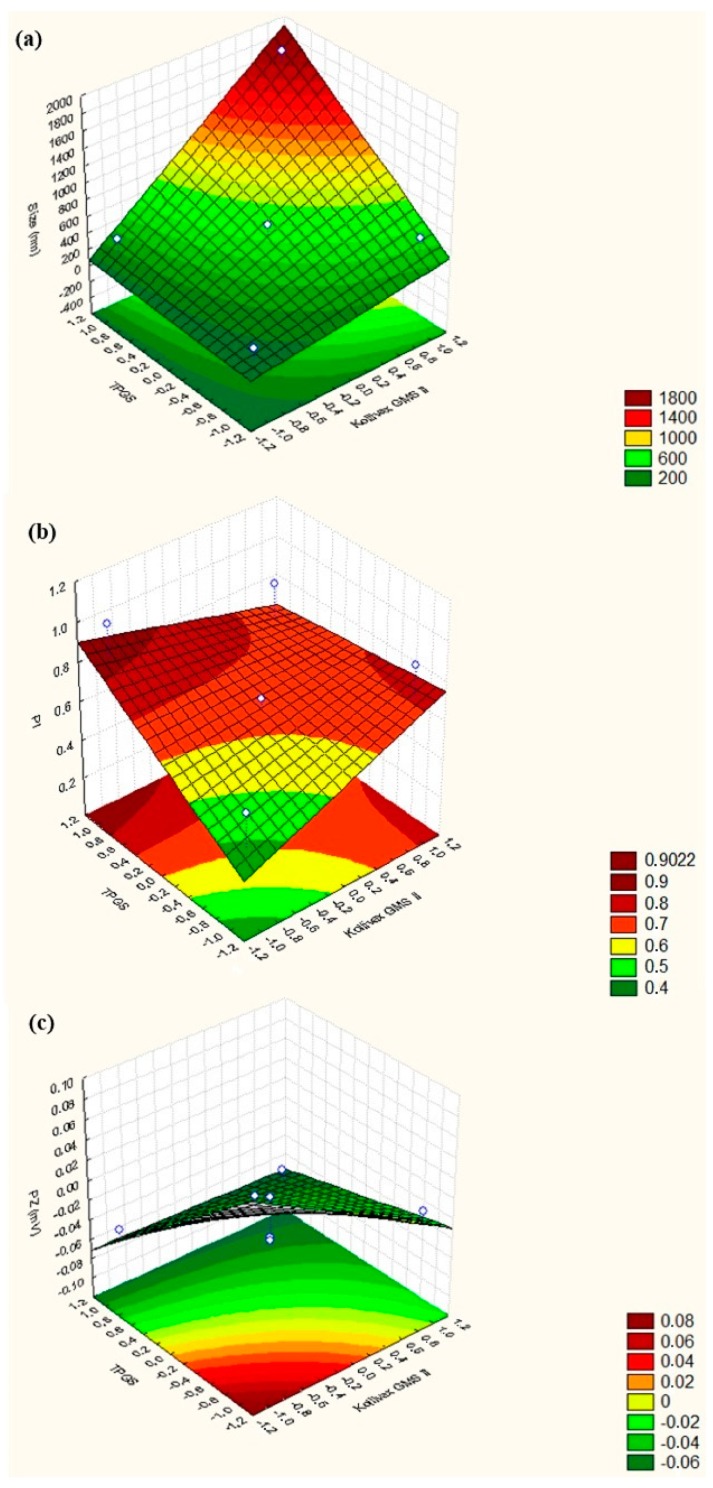
3D surface response chart showing the influence of the two factors (surfactant and lipid concentrations) on the (**a**) mean particle size, (**b**) PI, and (**c**) ZP of sucupira oil-loaded NLC.

Attempting to study interactive effects on different dependent variables, 3D surface response charts were used (Figure 2). Values referring to response alterations were plot in the Z-axis and the levels of the independent variables in X-axis (TPGS concentration) and Y-axis (Kollivax^®^ GMS II concentration). Increasing the surfactant concentration, particle size decreases to <200 nm (Figure 2a), PI increases up to 0.9 arb units (Figure 2b) and ZP also decreases (Figure 2c). On the other hand, when increasing solid lipid concentration, particle size increases up to 600 nm (Figure 2a), PI increases up to 0.9 arb units (Figure 2b) and ZP decreases (Figure 2c). According to our findings, an optimal sucupira oil-loaded NLC should consists of 0.5% (*m*/*V*) sucupira essential oil, 4.5% (*m*/*V*) of Kollivax^®^ GMS II and 1.425% (*m*/*V*) of TPGS. As such, since NLC no. 6 showed the best results and great stability, with a mean particle size ranging from 148.1 ± 0.9815 nm (0h) to 159.3 ± 9.539 nm (at 24 h), a PI ranging from 0.274 ± 0.029 (0 h) to 0.305 ± 0.028 (24 h) and a ZP close to zero (−0.00236 ± 0.147 mV at 0 h and 0.125 ± 0.162 at 24 h), it has been selected for cytotoxicity studies. 

The EE of sucupira oil-NLC (formulation no. 6) was found to be 99.98% and the LC 9.6%. These results were expected and were attributed to the high affinity of the oil with the solid lipid (Kollivax^®^ GMS II) selected by lipid screening assay. The obtained values are typical for oils loaded into lipid matrices and confirm the selection made from the factorial design experiment. As sucupira oil was shown to be soluble in the melted glycerol myristate (Dynasan 116), the selection of this solid lipid for the production of NLC resulted in the total encapsulation of the oil inside the lipid matrices. The loading capacity, translated by the ratio between sucupira oil and the solid lipid also confirmed their affinity both at high and at low temperatures (upon cooling), the oil remained entrapped inside NLC. 

**Figure 3:**
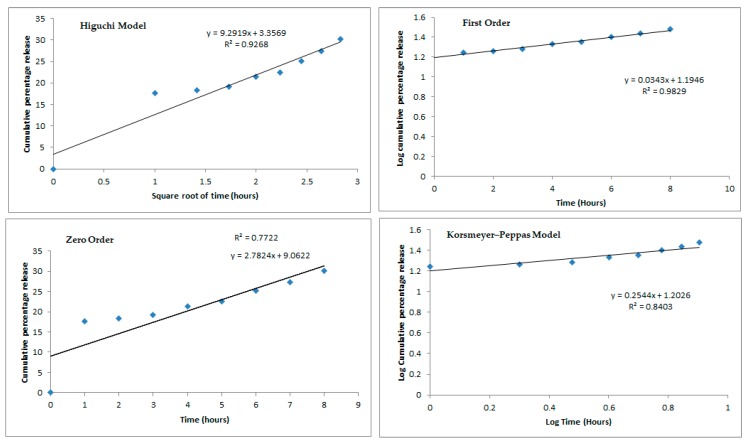
Mathematical fitting models (i.e. Higuchi Model, First Order, Zero Order and Korsemeyer-Peppas Model) of the release profile of sucupira oil from NLC (Formulation n °6) over 8 h.

Sucupira oil-NLC (formulation no. 6) was placed onto Franz diffusion cell for the recording of the release profile values, which were fitted into four mathematical models (Figure 3) [67]. The release profiles of sucupira oil from NLC followed a sustained fashion over the course of 8 h. Within the first hour only about 17.5% of oil has been released from NLC, whereas at the end of the 8 h the cumulative amount reached 30% of the loaded oil.

From the profiles shown in Figure 3, the best model describing the release of sucupira oil from NLC was the first order as *R*^2^ of 0.9829, the closest to 1. The first order release kinetics describes that variations in concentration over time only depends on the concentration of the sucupira oil inside the particles and is translated by the following equation:

(3) $$\frac{dM^{m}}{dt}=\;-kM^{m}$$

where $M^{m}$ is the concentration of sucupira oil in the NLC matrices and *k* is the first-order release constant. By integration, the logarithmic equation is obtained:

(4) $$ln\left(\frac{M_{0}^{m}}{M_{t}^{m}}\right)=kt$$

where $M_{0}^{m}$ is the initial concentration of sucupira oil in the NLC matrices and $M_{t}^{m}$ is the concentration of sucupira oil in the NLC matrices at time *t*. A modified release has been obtained which is not governed by the physicochemical properties of the carriers. These results were expected as NLC are not made of solid lipid only [37]. Indeed, Higuchi and Korsmeyers–Peppas are typically seen in the release profiles of SLN formulations as these have a higher degree of crystallinity (confirmed by DSC and X-ray) which controls the release profile. On the other hand, NLC have a lower degree of crystallinity as these latter also have a liquid lipid in their matrix. We have seen that it is the concentration of this liquid lipid (sucupira oil) which governs its release profile.

**Figure 4:**
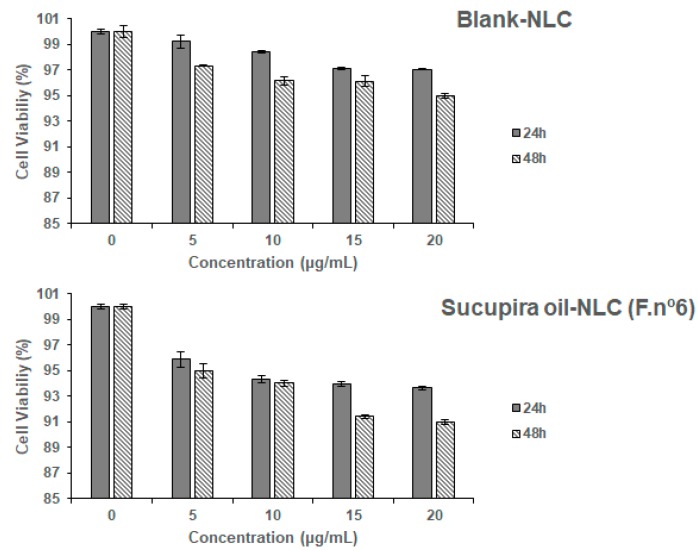
Caco-2 cell viability after 24 h and 48 h of exposure to different concentrations of Blank-NLC (upper panel) and sucupira oil-loaded NLC (lower panel). Results of cell viability are expressed as percentage of control cells (non-exposed cells) and as arithmetical means ± standard deviations (n = 3 different experiments). Cell viability was assayed by AlamarBlue^®^ reduction assay, for details see methods.

Blank NLC (i.e., without sucupira oil) and formulation no. 6 was subject of cytotoxicity analysis against Caco-2 cells (Figure 4).

The human intestinal Caco-2 cell line, a cell line derived from a colorectal adenocarcinoma, has a epithelial like-morphology and is commonly used as in vitro model for cytotoxicity screening of formulations intended for oral administration. In this work, the cell viability results, was tested in two time-points and using empty (blank) and loaded NLC, as recommended by Silva et al. [68,69]. Results (Figure 4) demonstrate that both blank NLC and NLC loading sucupira oil are considered non-toxic. Indeed, cellular viability was greater than 95% and 90%, for blank and loaded-NLC, respectively, for all concentrations and time-points, which translates a high biocompatibility in vitro of the developed particles [62,68,70]. The selected surfactant d-α-tocopheryl polyethylene glycol succinate, or vitamin E TPGS, has been approved by the Food and Drug Administration as a safe ingredient and is commonly used in pharmaceutical dosage forms [71]. Alpha-tocopheryl can also figure as oily phase in the composition of lipid nanoparticles [72]. It is important to bear in mind that the components of nanoparticles might be as toxic as the loaded bioactives [69]. In the present work, it is shown that the nanoparticle’s components did not damage this cell line, in the range of tested concentrations (5, 10, 15, and 20 µg/mL, selected according to the literature [62]) as cell viability, at the highest concentration and 48 h exposure is about 95% of control (Figure 4, upper panel). Within the first 24 h of exposure to sucupira oil-NLC, the decrease of cell viability was ~5% (highest difference recorded for sucupira oil-NLC at 20 µg/mL). The long-term exposure of the Caco-2 cells to TPGS-surfaced NLC did not compromise their viability. Indeed, after 48 h exposure, the cell viability was at 95% of control, for the blank-NLC, and at 91% of control for sucupira oil-NLC at the highest concentration (20 µg/mL). Taken altogether, this NLC formulation is considered non-toxic at the tested concentrations and time-points.

## 4. Conclusions

This work described the successful loading of sucupira oil in NLC composed of a new combination of Kollivax^®^ GMS II and of TPGS. A 2^2^ factorial design has been successfully used to determine the best combination of solid lipid and surfactant. An optimal sucupira oil-loaded NLC should consist of 0.5% (*m*/*V*) sucupira essential oil, 4.5% (*m*/*V*) of Kollivax^®^ GMS II and 1.425% (*m*/*V*) of TPGS, for which a mean particle size ranging from 148.1 ± 0.1 nm (0 h) to 159.3 ± 9.5 nm (at 24 h), a PI from 0.274 ± 0.029 (0 h) to 0.305 ± 0.028 (24 h) and a ZP from −0.00236 ± 0.147 mV (at 0 h) to 0.125 ± 0.162 (at 24 h) was recorded. The release profile followed the first order kinetics over the course of 8 h. For the optimized formulation an EE of 99.98% and LC of 9.6% were obtained. The particles also showed no cytotoxic effect when tested against Caco-2 cell line, up to 48 h. Further studies are planned to evaluate the interest of sucupira oil-NLC as an innovative anti-hyperglycemic formulation in a diabetic animal model. 

## Figures and Tables

**Table 1:** Composition of surfactant screening formulations.

Samples (V_T_ = 1 mL)	TPGS % (*m*/*V*)	Tween 80% (*m*/*V*)	Poloxamer^®^ 188 % (*m*/*V*)	Lecithin % (*m*/*V*)	Sucupira % (*m*/*V*)	Kollivax^®^ GMS II % (*m*/*V*)
1	4.5	1	1	0.5	-	-
2	4.5	1	1	-	-	-
3	4.5	2	-	0.5	-	-
4	4.5	2	-	-	-	-
5	4.5	-	2	0.5	-	-
6	4.5	-	2	-	-	-
7	-	0.5	-	-	0.5	4.5
8	-	1	-	-	0.5	4.5
9	-	1.5	-	-	0.5	4.5
10	4.5	1	1	-	0.5	-
11	-	1.5	-	-	0.5	-
12	-	1.5	-	0.5	0.5	-

**Table 2:** Composition of sucupira oil-loaded nanostructured lipid carrier (NLC) formulations.

Samples (V_T_ = 1 mL)	Sucupira Oil % (*m*/*V*)	Imwitor^®^ 900K % (*m*/*V*)	Kollivax^®^ GMS II % (*m*/*V*)	TPGS % (*m*/*V*)
1	0.5	4.5	-	0.475
2	0.5	4.5	-	0.950
3	0.5	4.5	-	1.425
4	0.5	-	4.5	0.475
5	0.5	-	4.5	0.950
6	0.5	-	4.5	1.425
7	0.75	4.25	-	0.475
8	0.75	4.25	-	0.950
9	0.75	4.25	-	1.425

**Table 3:** Initial 2-level full factorial design providing the lower (−1), upper (+1) and central point (0) level values for each variable.

Factors	Levels		
	−1	0	+1
Kollivax^®^ GMS II	2.250 % (*m*/*V*)	4.500 % (*m*/*V*)	9.00 % (*m*/*V*)
TPGS	0.7125 % (*m*/*V*)	1.425 % (*m*/*V*)	2.85 % (*m*/*V*)

**Table 4:** Composition of lipid screening samples that demonstrated no macroscopic phase separation, at 0 h, 1 h, and 24 h after their production.

Samples (V_T_ = 1 mL)	Sucupira Oil % (*m*/*V*)	Solid Lipid % (*m*/*V*)	Visual Analysis of Melted Mixtures			
			0 h	1 h	24 h	
1	5	Imwitor^®^ 900 K 95	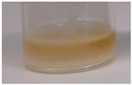	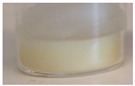	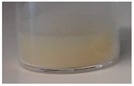	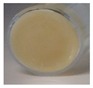
2	5	Dynasan^®^ 116 95	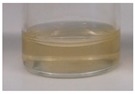	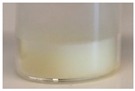	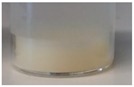	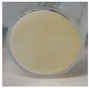
3	5	Kollivax^®^ GMS II 95	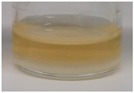	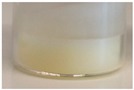	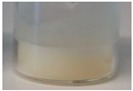	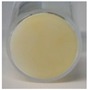
4	5	Cetostearyl alcohol 95	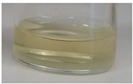	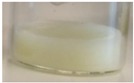	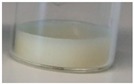	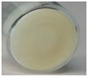
5	10	Imwitor^®^ 900 K 90	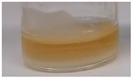	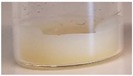	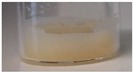	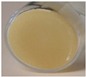
6	10	Dynasan^®^ 116 90	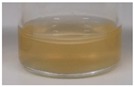	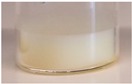	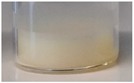	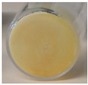
7	10	Kollivax^®^ GMS II 90	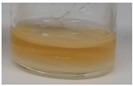	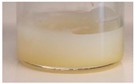	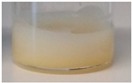	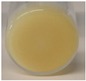
8	15	Imwitor^®^ 900 K 85	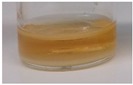	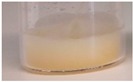	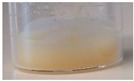	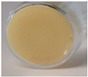
9	15	Kollivax^®^ GMS II 85	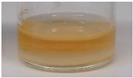	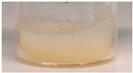	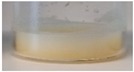	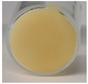

**Table 5:** Preliminary screening of surfactant based on the mean particle size, polydispersity index (PI) and zeta potential (ZP) of formulations without solid lipid (no. 1–6) and formulations with solid lipid (no. 7–12), measured immediately after production (0 h) and after 24 h stored at 4 °C.

Formulations (V_T_ = 1 mL)	Measurement Time (h)	Mean Particle Size (nm) ± SD	PI (arb. units) ± SD	ZP (mV) ± SD
1	0	197.8 ± 6.3	0.327 ± 0.008	−31.2 ± 1.7
	24	173.3 ± 2.9	0.656 ± 0.036	−28.7 ± 0.3
2	0	337.5 ± 122.9	0.367 ± 0.089	−7.0 ± 1.6
	24	21.4 ± 2.1	0.242 ± 0.053	−5.4 ± 1.4
3	0	201.8 ± 18.2	0.385 ± 0.078	−30.0 ± 0.7
	24	239.0 ± 8.7	0.546 ± 0.176	−28.6 ± 1.0
4	0	213.6 ± 58.7	0.274 ± 0.037	−13.8 ± 2.5
	24	63.6 ± 63.9	0.192 ± 0.041	−6.8 ± 2.1
5	0	208.2 ± 7.6	0.354 ± 0.100	−32.5 ± 1.2
	24	298.8 ± 17.4	0.528 ± 0.071	−32.6 ± 0.3
6	0	213.9 ± 89.1	0.334 ± 0.117	−0.32 ± 0.2
	24	152.7 ± 94.7	0.304 ± 0.074	−0.18 ± 0.3
7	0	957.1 ± 488.5	0.782 ± 0.206	−31.0 ± 1.1
	24	831.7 ± 129.3	0.728 ± 0.105	−26.4 ± 0.4
8	0	342.9 ± 87.8	0.645 ± 0.126	−30.1 ± 1.7
	24	1101.0 ± 275.9	0.860 ± 0.043	−23.8 ± 0.2
9	0	538.5 ± 117.6	0.520 ± 0.091	−28.0 ± 0.9
	24	812.9 ± 166.7	0.655 ± 0.086	−24.6 ± 0.6
10	0	443.8 ± 132.9	0.502 ± 0.082	−26.0 ± 1.4
	24	1189.0 ± 218.1	0.764 ± 0.025	−25.3 ± 0.5
11	0	833.7 ± 174.9	0.762 ± 0.128	−29.0 ± 0.4
	24	>1 µm	0.908 ± 0.086	−29.9 ± 0.2
12	0	243.6 ± 94.0	0.351 ± 0.063	−33.7 ± 0.4
	24	969.4 ± 301.2	0.813 ± 0.104	−27.7 ± 1.5

**Table 6:** Mean particle size, polydispersity index (PI) and zeta potential (ZP) of formulations without solid lipid (no. 1–6) and formulations with solid lipid (no. 7–12), measured immediately after production (0 h) and after 24 h stored at 4 °C, of Sucupira-loaded NLC.

Formulations (V_T_ = 1 mL)	Measurement Time (h)	Mean Particle Size (nm) ± SD	PI (arb. units) ± SD	ZP (mV) ± SD
1	0	147.2 ± 0.4	0.291 ± 0.025	-
	24	358.1 ± 2.2	0.660 ± 0.015	+0.02 ± 0.18
2	0	274.6 ± 10.6	0.645 ± 0.019	-
	24	190.6 ± 4.1	0.363 ± 0.016	−0.05 ± 0.07
3	0	118.0 ± 1.7	0.278 ± 0.008	-
	24	147.4 ± 2.3	0.337 ± 0.006	+0.12 ± 0.11
4	0	255.0 ± 8.4	0.526 ± 0.050	+0.05 ± 0.24
	24	318.8 ± 2.4	0.502 ± 0.001	+0.05 ± 0.16
5	0	164.8 ± 0.7	0.379 ± 0.020	−0.14 ± 0.11
	24	178.1 ± 1.0	0.298 ± 0.026	−0.04 ± 0.14
6	0	148.1 ± 1.0	0.274 ± 0.029	−0.15±0.002
	24	159.3 ± 9.5	0.305 ± 0.028	+0.13 ± 0.16
7	0	752.2 ± 752.5	0.602 ± 0.249	−0.04 ± 0.06
	24	244.5 ± 0.9	0.264 ± 0.007	−0.07 ± 0.16
8	0	136.2 ± 2.0	0.242 ± 0.009	−0.04 ± 0.01
	24	272.2 ± 1.4	0.498 ± 1.353	+0.02 ± 0.1
9	0	117.9 ± 0.7	0.267 ± 0.021	+0.01 ± 0.07
	24	202.1 ± 2.1	0.398 ± 0.023	−0.09 ± 0.08

**Table 7:** 2^2^ full factorial design for the development of sucupira-oil loaded NLC and respective response parameters.

NLC Formulation (V_T_ = 50 mL)	Independent Variables		Dependent Variables		
	Kollivax^®^ GMS II	TPGS	Particle Size (nm) ± SD	PI (arb units) ± SD	ZP (mV) ± SD
1	0	0	344.2 ± 39.43	0.0613 ± 0.066	−0.0512 ± 0.0134
2	0	0	604.7 ± 83.64	0.666 ± 0.059	−0.00793 ± 0.112
3	0	0	212.4 ± 9.158	0.567 ± 0.057	−0.0483 ± 0.00758
4	+1	+1	1762 ± 225.8	0.832 ± 0.146	−0.0604 ± 0.0290
5	+1	−1	537.5 ± 13.78	0.873 ± 0.094	−0.0143 ± 0.0837
6	−1	−1	180.7 ± 11.82	0.553 ± 0.086	+0.0757 ± 0.0425
7	−1	+1	337.6 ± 67.88	1.000 ± 0.000	−0.0486 ± 0.0517

**Table 8:** Analysis of the mean particle size by ANOVA statistical test. Determination coefficient (R^2^) = 0.86039.

Mean Particle Size					
Factors and Interactions	Sum of Squares (SS)	Degrees of Freedom (df)	Mean Square (MS)	F-Value	*p*-Value
(1) Kollivax^®^ GMS II	793,168	1	793,168.4	9.429096	0.054533
(2) TPGS	477,066	1	477,066.5	5.671312	0.097490
1 by 2	284,942	1	284,942.4	3.387363	0.162690
Error	252,358	3	84,119.2		
Total	1,807,535	6			

**Table 9:** Analysis of the mean polydispersity index by ANOVA statistical test. Determination coefficient (R^2^) = 0.18738.

Mean Polydispersity Index					
Factors and Interactions	Sum of Squares (SS)	Degrees of Freedom (df)	Mean Square (MS)	F-Value	*p*-Value
(1) Kollivax^®^ GMS II	0.005776	1	0.005776	0.037510	0.858801
(2) TPGS	0.041209	1	0.041209	0.267618	0.640659
1 by 2	0.059536	1	0.059536	0.386637	0.578125
Error	0.461953	3	0.153984		
Total	0.568474	6			

**Table 10:** Analysis of the mean zeta potential by ANOVA statistical test. Determination coefficient (R^2^) = 0.84107.

Mean Zeta Potential					
Factors and Interactions	Sum of Squares (SS)	Degrees of Freedom (df)	Mean Square (MS)	F-Value	*p*-Value
(1) Kollivax^®^ GMS II	0.002591	1	0.002591	3.61478	0.153441
(2) TPGS	0.007259	1	0.007259	10.12803	0.050000
1 by 2	0.001529	1	0.001529	2.13304	0.240284
Error	0.002150	3	0.000717		
Total	0.013529	6			
